# Nitric oxide facilitates the S‐nitrosylation and deubiquitination of Notch1 protein to maintain cancer stem cells in human NSCLC

**DOI:** 10.1111/jcmm.70203

**Published:** 2024-11-10

**Authors:** Tenglong Zhang, Jiaxin Lei, Ming Zheng, Zhenke Wen, Juying Zhou

**Affiliations:** ^1^ Department of Radiation Oncology The First Affiliated Hospital of Soochow University, Soochow University Suzhou China; ^2^ Department of Oncology Qingdao Municipal Hospital Qingdao China; ^3^ The Fourth Affiliated Hospital of Soochow University, Institutes of Biology and Medical Sciences, Suzhou Medical College of Soochow University Soochow University Suzhou China; ^4^ Jiangsu Key Laboratory of Infection and Immunity, MOE Key Laboratory of Geriatric Diseases and Immunology, Suzhou Medical College of Soochow University Soochow University Suzhou China

**Keywords:** cancer stem cell, Notch1, NSCLC

## Abstract

Non‐small cell lung cancer (NSCLC) is the leading cause of cancer‐related mortality, with tumour heterogeneity, fueled by cancer stem cells (CSCs), intricately linked to treatment resistance. Therefore, it is imperative to advance therapeutic strategies targeting CSCs in NSCLC. In this study, we utilized RNA sequencing to investigate metabolic pathway alterations in NSCLC CSCs and identified a crucial role of nitric oxide (NO) metabolism in governing CSC stemness, primarily through modulation of the Notch1 protein. Mechanistically, NO‐induced S‐nitrosylation of Notch1 facilitated its interaction with the deubiquitylase UCHL1, leading to increased Notch1 protein stability and enhanced CSC stemness. By inhibiting NO synthesis and downregulating UCHL1 expression, we validated the impact of NO on the Notch signalling pathway and CSC stemness. Importantly, targeting NO effectively reduced CSC populations within patient‐derived organoids (PDOs) during radiotherapy. This mechanism presents a promising therapeutic target to surmount radiotherapy resistance in NSCLC treatment.

## 1 INTRODUCTION

Non‐small‐cell lung cancer (NSCLC) is one of the leading causes of cancer‐related deaths worldwide, characterized by a notably low survival rate and a high tendency for metastasis to organs such as the brain, skeleton and liver.^1^, ^2^ The treatment of NSCLC faces substantial hurdles, largely attributed to the central role of cancer stem cells (CSCs), which drive disease initiation, advancement, recurrence and therapeutic resistance.^3^, ^4^ Consequently, targeting NSCLC CSCs holds promise to counteract treatment resistance and improve prognosis. CSCs undergo asymmetric division to maintain CSC pool and differentiate into various tumour cell types, thereby promoting intra‐tumour heterogeneity. Consequently, CSCs are regarded as the ‘seed cells’ for tumorigenesis.^5^ Aberrations in signalling pathways such as Notch, Hedgehog and Wnt^6^, ^7^ within CSCs have profound effects on cell proliferation, differentiation and apoptosis, positioning them as key therapeutic targets. Notably, the Notch signalling pathway plays a pivotal role in promoting self‐renewal, differentiation, proliferation, survival and migration of CSCs in various cancers, including lung cancer.^6^, ^7^, ^8^ Activation of Notch signalling fuels solid tumour progression by orchestrating angiogenesis and maintaining an undifferentiated CSC pool.^6^ Further, while the genes linked to CSCs may vary across cancer types, markers such as SOX2, OCT4, ALDH1 and CD133, are typically expressed on CSCs across diverse malignancies.^9^ These genes serve as reliable indicators of CSC stemness in different cancer contexts.^3^, ^10^, ^11^

The metabolism of CSCs is increasingly recognized as a critical aspect of CSC biology. In contrast to differentiated bulk tumour cells, which predominantly rely on glycolysis, CSCs exhibit a distinct metabolic phenotype that can depend either on glycolysis or oxidative phosphorylation (OXPHOs).^3^, ^12^ Of interest, the multifaceted biological roles of NO metabolism in CSC stemness have increasingly garnered attention in recent years.^13^, ^14^ The impact of NO on tumour biology is recognized to be contingent upon its source, timing, spatial distribution and concentration levels. Studies have revealed that inducible nitric oxide synthase (iNOS) can stimulate the Notch signalling pathway in liver cancer stem cells, bolstering their stemness and accelerating liver cancer advancement.^13^ Nonetheless, the potential involvement of NO metabolism in regulating the stemness of NSCLC CSCs remains largely unexplored.

In this study, we delved into the metabolic characteristics of NSCLC CSCs, highlighting NO metabolism as a key aspect. Significantly, we discovered that NO maintains CSC stemness by fostering S‐nitrosylation of Notch1 protein, leading to an augmentation in its deubiquitination. By targeting NO metabolism, we demonstrated a significant reduction in CSC abundance within NSCLC patient‐derived organoids (PDOs) and an enhancement in their radiosensitivity, offering a promising avenue for clinical NSCLC treatment.

## 2 PATIENTS AND METHODS

### 2.1 Patients

Patients diagnosed with NSCLC adenocarcinoma were recruited, while those with other uncontrolled medical diseases or inflammatory diseases were excluded. Patients' characteristics, including gender, age, and TNM stages, were summarized in Table S1. Appropriate informed consent was obtained from every individual before collecting the clinical samples, and all studies were approved by the Ethics Committee of Soochow University.

### 2.2 Patient‐derived organoids

Patient‐derived organoids (PDOs) were established and cultured as previously described.^15^ Briefly, NSCLC tissues from clinical patients were minced with fine dissection scissors (Fine Science Tools) until approximately 0.5–1 mm diameter pieces. After that, RBC lysis buffer was added to the minced tissue to lyse contaminating RBCs. Then, minced tissues were resuspended with 4 mL per well of PDO medium containing 50% DMEM/F12 (Corning), 50% Neurobasal (Thermo Fisher Scientific), 1× GlutaMax (Gibco), 1× NEAAs (Thermo Fisher Scientific), 1× PenStrep (Beyotime), 1× N2 supplement (Thermo Fisher Scientific), 1X B27 w/o vitamin A supplement (Thermo Fisher Scientific), 1 × 2‐mercaptoethanol (Thermo Fisher Scientific), 2.5 mg/mL human insulin (Beyotime), distributed in ultralow attachment six‐well culture plates (Corning), and placed on an orbital shaker rotating at 120 rpm within a 37°C, 5% CO_2_, 90% humidity sterile incubator. The fresh PDO medium was changed approximately twice a week. Such PDOs were assessed via immunostainings of PanCK (Abcam, ab7753) and CD31 (Abcam, 281583).

### 2.3 Tumour spheres and adherent cells

Tumour spheres were enriched and used as CSCs as previously described.^3^ Human NSCLC cell line A549 purchased from ATCC were cultured in DMEM with 10% FBS (PAN Seratech). For CSCs enrichment, A549 cells were resuspended at a density of 5,000 cells/mL with serum‐free DMEM/F12 medium containing B27 supplement, insulin (4 μg/mL), EGF (20 ng/mL), and FGF (20 ng/mL) in low‐attachment six‐well plates (Corning). The adherent cells were used as non‐CSCs.

## 3 REAGENTS AND TRANSFECTION

### 3.1 Reagents

L‐NAME, 1400 W 2HCl and S‐Nitrosoglutathione (GSNO) was purchased from Selleck (L‐NAME, S2877; 1400 W, S8337; GSNO, E4443) and DETA NONOate, Mitomycin C and proteasome inhibitor MG132 were from MCE (DETA NONOate, HY‐136278; Mitomycin C, HY‐13316; MG132, HY‐13259). The sGC inhibitor ODQ was from Topscience (T19777).

### 3.2 Gene silencing and overexpression

Human UCHL1 shRNA, WWP1 shRNA, MDM2 shRNA, USP9X shRNA and PSMD7 shRNA was purchased from Tsingke. Human Notch1 intracellular domain plasmid (#130934) was from Addgene. Human Myc‐ubiquitin was from MiaoLing Plasmid Platform. CSCs and non‐CSCs transfections were performed using the Lipomaster 3000 Transfection Reagent (Vazyme). All reagents were used according to the manufacturers' instructions.

### 3.3 Quantitative PCR

Quantitative PCR (qPCR) was performed as previously described.^16^, ^17^ Briefly, cells were harvested with Trizol (TAKARA) reagent. The cDNA was produced by reverse transcription using RT SuperMix (Vazyme) according to the manufacturer's instructions using total of 1 mg of RNA. Quantitative PCR analyses were carried out using SYBR Master Mix (Bimake). The relative quantity of mRNA was expressed as 2−^ΔCt^. Sequences of the primers were listed in Table S2.

### 3.4 RNA sequencing

Tumour spheres and adherent cells underwent RNA sequencing (RNA‐Seq) analysis conducted by the GIGA Genomics Facility (BioNovoGene).^3^ This data has been deposited in the National Genomics Data Center under accession no. PRJCA006881. For RNA‐Seq library preparation, 1–2 μg of total RNA from each sample was utilized, employing the KAPA Stranded RNA‐Seq Library Prep Kit (Illumina). Raw sequencing reads underwent initial processing, including adapter sequence trimming, and filtering based on a minimum average quality score of Q20 to generate high‐quality clean data. Subsequently, HISAT2 was employed with default parameters for alignment of the clean sequencing reads to the human reference genome. HTSeq was utilized to quantify gene expression levels based on read counts per gene. Differential expression analysis was conducted using DESeq, a robust tool for RNA‐Seq data analysis. Genes were deemed significantly differentially expressed if they exhibited an absolute fold change exceeding 2 and an adjusted *p* value below 0.05.

### 3.5 Measurement of intracellular cGMP level

CSCs were cultured in 24‐well plates for 24 h with DETA NONOate, GSNO, L‐NAME and 1400 W. Following the repeated freeze–thaw process, the cell lysates were centrifuged, and the supernatants were collected for the assay. The concentration of cGMP was determined using the cGMP ELISA Kit (Elabscience). Subsequently, the results were quantified employing the Synergy™ H4 Hybrid Reader (BioTek Instruments) at a wavelength of 450 nm.

### 3.6 Wound healing assay

A549 cells were cultured in the presence or absence of DETA NONOate for 24 h. Subsequently, a scratch was introduced into the cellular monolayer using a sterile pipette tip. After PBS washing, cells were incubated in a complete medium containing Mitomycin C at 37°C for 1 h. After another PBS wash, the medium was replaced with one containing 2% FBS for continued culture. Pictures of per well were captured immediately after the medium change and subsequently at 12 and 24‐h intervals. The percentage of wound healing was analysed using ImageJ software.

### 3.7 Immunoblotting and immunoprecipitation

Experimental conditions for immunoblotting and immunoprecipitation experiments have been previously described.^8^, ^16^ The antibodies with dilutions were as follows: anti‐β‐actin (Santa Cruz Biotechnology, sc‐47778, 1:1000), anti‐Notch1 (Cell Signalling Technology, D1E11, 1:1000), anti‐Hes1 (Cell Signalling Technology, D6P2U, 1:1000), anti‐UCHL1 (Proteintech, 14730‐1‐AP, 1:1000), anti‐Ubiquitin (Santa Cruz Biotechnology, sc‐8017, 1:500), anti‐Myc Tag (Abmart, 19C2, 1:3000).

### 3.8 Biotin‐switching assay

SNO‐Notch1 level were assessed by biotin switch assay with the S‐nitrosylation Protein Detection Assay Kit (Cayman, 10006518) as described previously.^18^, ^19^ In brief, the free thiols in cell lysates were blocked with blocking buffer for 30 min and the proteins were precipitated with cold acetone. Then, the samples were incubated with reducing buffer and labeling buffer, converting the S‐nitrosothiols to free thiols and labeling them with biotin. After continuous rotation, the biotinylated proteins were collected with streptavidin agarose (Beyotime, P2159), followed by SDS‐PAGE analysis with anti‐Notch1 antibody.

### 3.9 Flow cytometry

Flow cytometry was performed as previously described.^16^, ^17^ Cells from PDOs were prepared using 300 units/mL collagenase IV (Worthington, LS004210) at 37°C for 2 h, followed by three 5‐min washes with RPMI‐1640 (Corning) supplemented with 10% FBS. Cells were treated with BD Fix Buffer I and Cytoperm Buffer and were stained with the following antibodies: PE‐conjugated anti‐CD133 antibody (Invitrogen, 12‐1338‐42), anti‐human Notch1 antibody (Santa Cruz Biotechnology, sc‐373891) plus goat anti‐mouse IgG (DyLight 649, Abbkine, A23610). Cells were stained for 30 min at room temperature in the dark, followed by thorough washing and flow cytometry analysis using a Canto II (BD Biosciences). Data were analysed with the FlowJo software.

### 3.10 Immunofluorescence

Immunofluorescence staining was performed as previously described.^20^ Tissue slides were fixed in ice‐cold acetone for 20 min, then washed with 1× PBS. Next, tissue sections were incubated overnight at 4°C with anti‐CD31(Abcam, ab281583) and anti‐PanCK (Abcam, ab7753). After washing, slides were incubated with secondary goat anti‐mouse IgG (DyLight 649, Abbkine, A23610) and goat anti‐rabbit IgG (Dylight 488, Abbkine, A23220) for 1 h. Nuclei were counterstained with Hoechst 33342 (Yeasen, 40731ES10) for 15 min at room temperature. Images were visualized with confocal microscopy (Nikon) and deconvolved with ImageJ software (NIH).

### 3.11 Radiation delivery in vitro

NSCLC PDOs are placed in 24‐well low‐attachment plates and irradiated at a dose of 10 Gy per min using RS‐2000 Pro (Rad source, USA).

### 3.12 Statistics

Data were presented as the mean ± SEM. Paired and unpaired student *t*‐tests were used for two‐group comparisons as appropriate. One‐way ANOVA with the Turkey method was used for more than two‐group comparisons. All statistical analyses were conducted using PRISM 9.5 (GraphPad Software Inc.), and *p* < 0.05 was considered significant. The number of independent experiments or individuals was provided in each figure legend.

## 4 RESULTS

### 4.1 Higher level of NO licences stem‐like properties of CSCs

**FIGURE 1:**
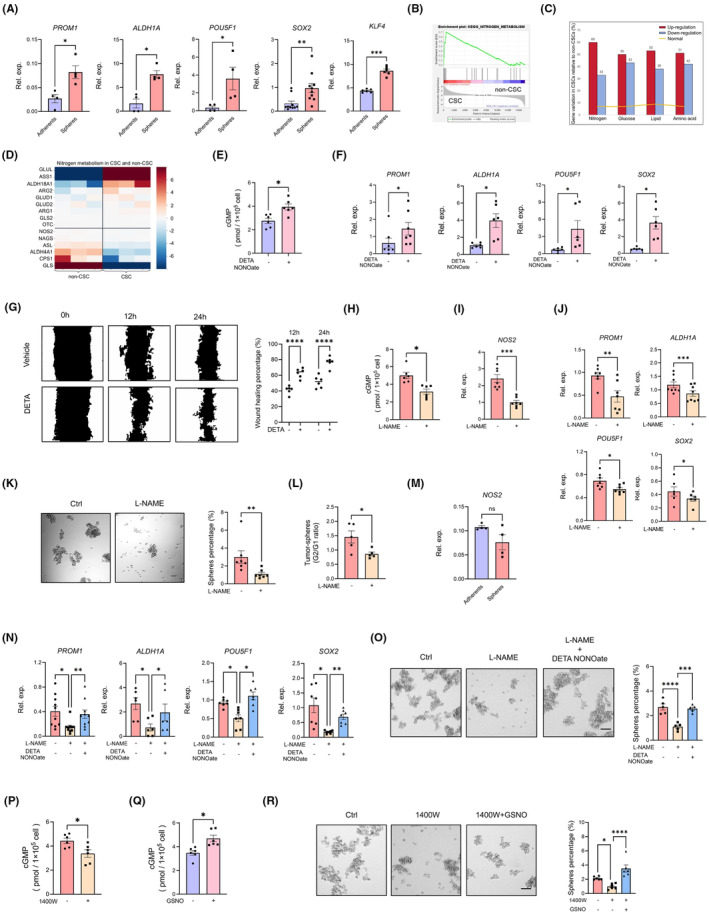
NO preserves the stemness of CSCs. (A) mRNA expressions of PROM1, ALDH1, POU5F1, SOX2 and KLF4 were assessed in non‐CSCs and CSCs by qPCR. Mean ± SEM from 4 to 10 independent experiments. (B) GSEA snapshots of KEGG pathway enrichment analysis. (C) Genes involved in nitrogen, glucose, lipid, and amino acid metabolism were categorized into three groups, up‐regulation, down‐regulation and no regulation in CSC compared to non‐CSC. (D) Heatmap showed nitrogen metabolism‐related genes expressed in CSCs compared with non‐CSCs. (E) non‐CSCs were treated with DETA NONOate for 24 h and the concentrations of intracellular cGMP were determined by ELISA. (F) Non‐CSCs were treated with or without DETA NONOate (20 μM) for 24 h and detected for PROM1, ALDH1, POU5F1 and SOX2 mRNA expressions by qPCR. Mean ± SEM from 6 to 7 independent experiments. (G) Non‐CSCs were treated with or without DETA NONOate (20 μM) for 24 h and the wound healing assays were performed. Mean ± SEM from six independent experiments. (H) CSCs were treated with DETA NONOate for 24 h and the concentrations of intracellular cGMP were determined by ELISA. (I) CSCs treated with L‐NAME (100 μM) for 24 h were detected for expression of NOS2 by qPCR. Mean ± SEM from seven independent experiments. (J) mRNA levels of PROM1, ALDH1, POU5F1 and SOX2 were measured in CSCs with or without L‐NAME (100 μM) treatment for 24 h. Mean ± SEM from 6 to 8 independent experiments. (K) CSCs treated with or without L‐NAME (100 μM) for 7 days were detected for sphere formations. Representative images and Mean ± SEM from seven independent experiments were shown. Scale bar: 10 μm. (L) Ration of CSCs formation at first (G1) and second (G2) generation after L‐NAME (100 μM) treatment. Mean ± SEM from five independent experiments. (M) mRNA expression of NOS2 was assessed in non‐CSCs and CSCs by qPCR. Mean ± SEM from four independent experiments. (N) CSCs were exposed to L‐NAME (100 μM) in the presence or absence of the DETA NONOate (20 μM) for 24 h. mRNA expressions of indicated stem‐related genes were detected by qPCR. (O) CSCs conditioned with L‐NAME (100 μM), with or without the addition of DETA NONOate (20 μM), were assayed for sphere formation. Representative images and mean ± SEM from five independent experiments are shown. (P, Q) CSCs were treated with GSNO (200 μM) and 1400 W (100 μM) for 24 h and the concentrations of intracellular cGMP were determined by ELISA. (R) CSCs treated with 1400 W (100 μM) in the presence or absence of the GSNO (200 μM) were analysed for sphere formation. Mean ± SEM from 6 independent experiments. *p < 0.05, **p < 0.01, ***p < 0.001 and ****p < 0.0001 with paired t‐test (E, F, H–L, P, Q), unpaired *t*‐test (A, M) and ANOVA plus Turkey's method (G, N, O, R).

The qPCR assay was employed to assess the expression level of stemness markers, including PROM1, SOX2, POU5F1, ALDH1A and KLF4. Our results showed that the expression of stemness‐related genes enhanced significantly in NSCLC CSCs compared to non‐CSCs (Figure 1A). This observation indicates the effective enrichment of CSCs from the non‐CSC population. RNA sequencing was conducted for comparative analysis between CSC and non‐CSC populations. KEGG pathway analysis identified nitrogen metabolism signalling as the most prominently enhanced pathway in CSCs compared to non‐CSCs (Figure 1B–D). To substantiate the pivotal role of NO in promoting CSC stemness, we assessed the expression levels of stemness markers in non‐CSCs following treatment with the NO donor DETA NONOate. Upon confirming the efficacy of DETA NONOate (Figure 1E), we observed a significant upregulation in the expression levels of multiple stemness markers, (Figure 1F). Besides, DETA NONOate treatment significantly enhanced the migration of A549 cells (Figure 1G). These results suggested that elevated NO levels may promote the stemness of CSCs. Correspondingly, upon treatment with NOS inhibitor L‐NAME (Figure 1H,I), a significant decrease in the expression levels of stemness markers was observed in CSCs (Figure 1J). Furthermore, L‐NAME notably impaired the spheres‐forming ability of CSCs (Figure 1K,L). However, there was no difference in NOS2 expression between CSCs and non‐CSCs (Figure 1M). Additionally, DETA NONOate efficiently rescued the L‐NAME‐induced downregulation of stemness‐related transcripts (Figure 1N) and spheres‐forming ability of CSCs (Figure 1O). Comparable results were obtained following treatment with another NOS inhibitor, 1400 W, and the NO donor GSNO (Figure 1P–R). Collectively, these findings suggest that elevated NO levels play a crucial role in maintaining the stemness of LCSCs.

### 4.2 NO promotes the CSC phenotype via upregulating Notch1

**FIGURE 2:**
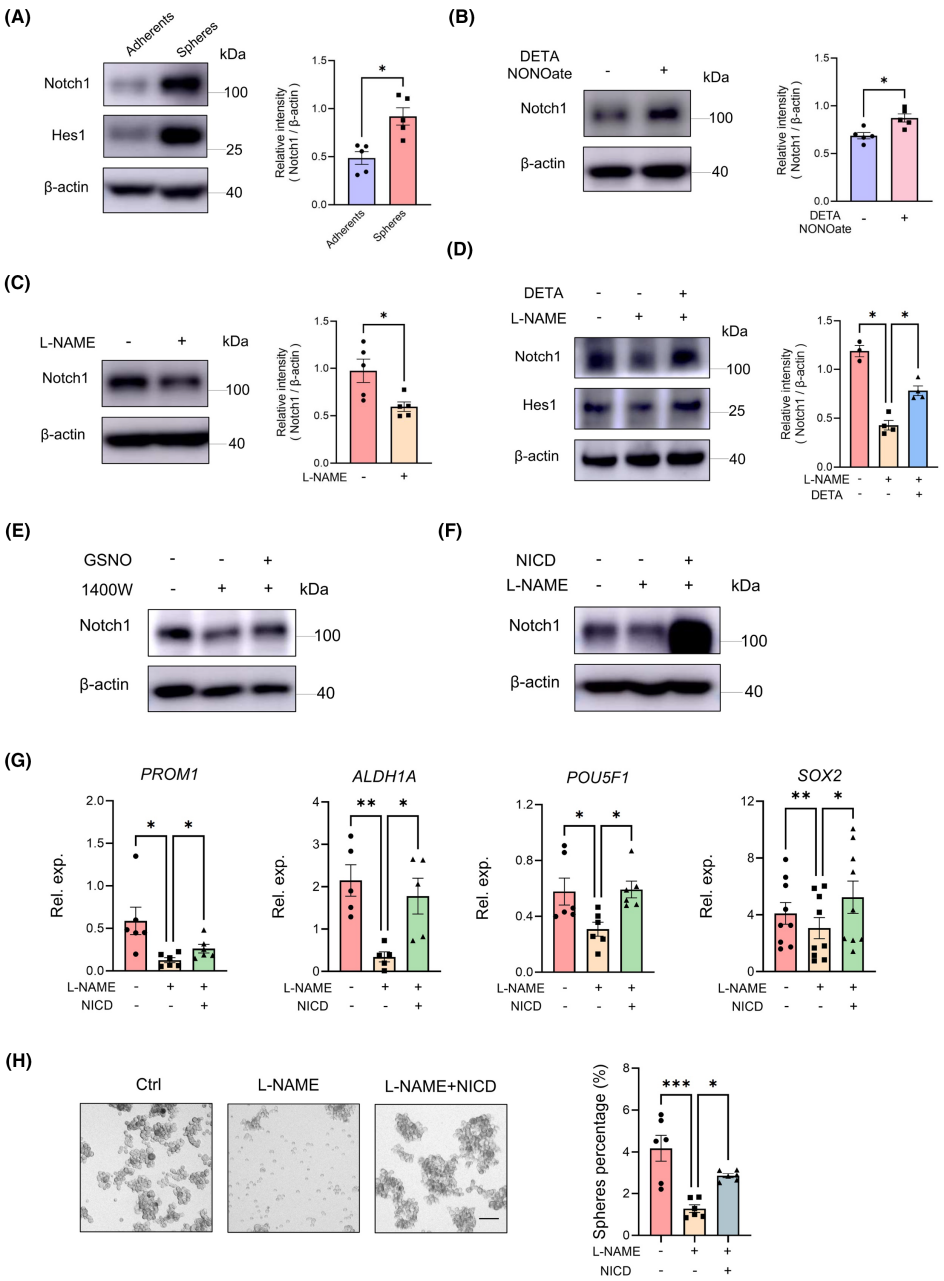
NO sustains CSC stemness through the activation of Notch1. (A) Immunoblot analysis of Notch1 and Hes1 protein in non‐CSCs and CSCs. (B) The protein expression of Notch1 was analysed in non‐CSCs treated with DETA NONOate (20 μM) for 24 h. (C) CSCs were treated with L‐NAME for 24 h and detected for Notch1 protein with immunoblots. (D, E) CSCs were treated with L‐NAME (100 μM)/1400 W (100 μM) in the presence or absence of the DETA NONOate (20 μM)/GSNO (200 μM) for 24 h. Protein expression of Notch1 and Hes1 were detected by immunoblot. (F–H) CSCs were exposed to L‐NAME (100 μM) with or without the reintroduction of NICD and were analysed for expression of Notch1 protein, stem‐related transcripts and spheres formation. Mean ± SEM from 5 to 9 independent experiments. *p < 0.05, **p < 0.01 and ***p < 0.001 with paired *t*‐test (B, C), unpaired *t*‐test (A) and ANOVA plus Turkey's method (D, G, H).

Notch1 is essential for the stemness and expansion of NSCLC CSCs.^8^ To elucidate whether Notch1 is implicated in NO‐mediated regulation of CSC stemness, we compared Notch1 protein levels in CSCs with that in non‐CSCs. Our findings revealed a significant upregulation in the expression levels of Notch1 and its downstream target protein, Hes1, in CSCs (Figure 2A). After DETA NONOate treatment, Notch1 protein levels in non‐CSCs exhibited a significant increase (Figure 2B), while L‐NAME treatment significantly inhibited Notch1 expression (Figure 2C). Notably, NO donors remarkably reversed the downregulation of Notch1 and Hes1 induced by NOS inhibitor (Figure 2D,E). Of importance, enforced expression of the Notch intracellular domain (NICD), a functional fragment of the Notch signal derived from γ‐secretase cleavage, counteracted the inhibitory effect of L‐NAME on CSC stemness, resulting in an increased level of Notch1 protein, elevated mRNA of stemness‐related transcripts and enhanced spheres‐forming ability (Figure 2F–H). These findings underscored the crucial role of NO in maintaining the stemness of LCSCs by elevating Notch1 protein levels.

### 4.3 NO drives deubiquitination of Notch1 in CSCs

**FIGURE 3:**
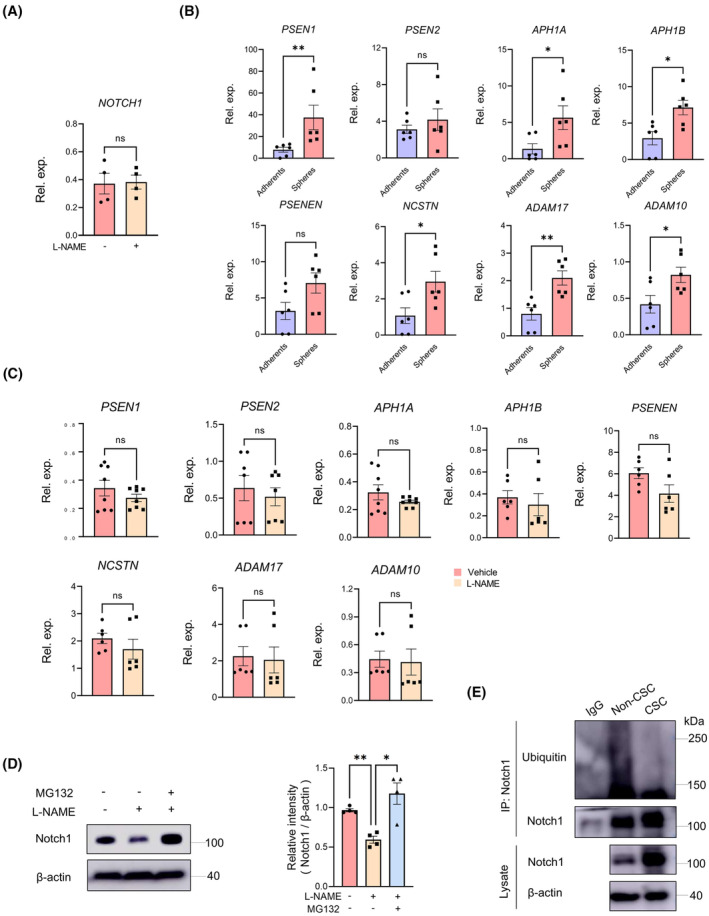
NO inhibits the ubiquitination of Notch1. (A) mRNA level of Notch1 was measured in CSCs with or without L‐NAME (100 μM) treatment for 24 h. Mean ± SEM from four independent experiments. (B) Notch1 cleavage‐related mRNA expressions in non‐CSCs and CSCs was determined with qPCR. Mean ± SEM from six independent experiments. (C) CSCs were treated with or without L‐NAME (100 μM) for 24 h and detected for Notch1 cleavage‐related mRNA expressions by qPCR. Mean ± SEM from 6 to 8 independent experiments. (D) CSCs were treated with L‐NAME (100 μM) in the presence or absence of the MG132 (10 μM) for 4 h. Protein expression of Notch1 was detected by immunoblot. Mean ± SEM from four independent experiments. (E) Notch1 ubiquitination in non‐CSCs and CSCs was analysed using endogenous immunoprecipitated Notch1 protein. *p < 0.05, **p < 0.01 with paired *t*‐test (A, C), unpaired *t*‐test (B) and ANOVA plus Turkey's method (D).

To define the mechanism underlying the upregulation of Notch1 protein by NO, we investigated the mRNA levels of Notch1 following L‐NAME treatment. Intriguingly, L‐NAME treatment had no effect on the mRNA level of the Notch1 in CSCs (Figure 3A). This suggested that NO‐mediated regulation of Notch1 expression did not occur at the transcriptional level.

It is widely acknowledged that Notch signalling is initiated by two distinct proteolytic events: ADAM‐mediated S2 cleavage and γ‐secretase‐mediated release of the NICD.^21^, ^22^ To elucidate potential disparities in Notch1 processing between CSCs and non‐CSCs, we initially examined the expression of cleaving enzymes involved in Notch1 protein processing. We observed a significant increase in the expression of cleaving enzymes in CSCs compared to non‐CSCs (Figure 3B). However, L‐NAME treatment did not affect the expression of these enzymes (Figure 3C), suggesting that the upregulation of Notch1 protein by NO is not associated with Notch1 protein processing.

Subsequently, we tested whether the ubiquitin‐proteasome pathway contributed to the L‐NAME‐mediated reduction in Notch1 protein levels. Remarkably, treatment with the proteasome inhibitor MG132 effectively restored the levels of Notch1 protein in L‐NAME‐pretreated CSCs (Figure 3D). This implied that L‐NAME promoted the ubiquitination of Notch1 protein in LCSCs, suggesting that reduced levels of NO led to Notch1 ubiquitination. In line with these observations, we noted a significant decrease in the ubiquitination of immunoprecipitated Notch1 in CSCs (Figure 3E). These results confirmed that NO suppressed the ubiquitination of Notch1 protein.

### 4.4 NO promotes the interaction of Notch1 to a deubiquitinase UCHL1

**FIGURE 4:**
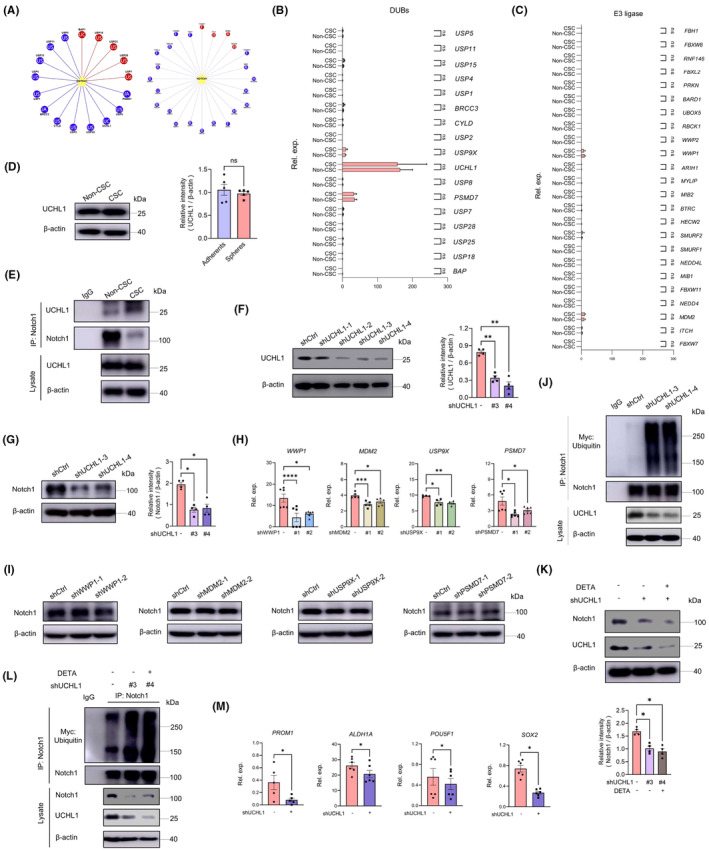
NO inhibits the ubiquitination of Notch1 by facilitating its interaction with UCHL1. (A) Potential DUBs and E3 ligase of Notch1 obtained from the UbiBrowser database. (B, C) DUBs and E3 ligase mRNA expressions in non‐CSCs and CSCs were determined with qPCR. Mean ± SEM from six independent experiments. (D) Immunoblot analysis of UCHL1 protein in non‐CSCs and CSCs. Mean ± SEM from five independent experiments. (E) Co‐immunoprecipitation analysis of Notch1‐UCHL1 interaction in non‐CSCs and CSCs. (F) Genetic knockdown efficiency of UCHL1 in CSCs by lentiviral shRNA transfections. Mean ± SEM from four independent experiments. (G) Immunoblot analysis of Notch1 protein in CSCs transfected with or without the independent UCHL1 shRNAs. Mean ± SEM from four independent experiments. (H) Genetic knockdown efficiency of WWP1, MDM2, USP9X and PSMD7 in CSCs by lentiviral shRNA transfections. Mean ± SEM from 4 to 6 independent experiments. (I) Immunoblot analysis of Notch1 protein in CSCs transfected with or without the independent WWP1, MDM2, USP9X and PSMD7 shRNAs. (J) Immunoprecipitation analysis of ubiquitination of Notch1 in CSCs that were transfected with shUCHL1. (K) CSCs were transfected with UCHL1 shRNAs in the presence or absence of the DETA NONOate (20 μM). The protein of Notch1 was analysed with immunoblot. Mean ± SEM from four independent experiments. (L) Immunoprecipitation analysis of Notch1 ubiquitination in CSCs that were transfected with UCHL1 shRNAs together with DETA NONOate (20 μM). (M) CSCs transfected with UCHL1 shRNAs were analysed for expression of stem‐related transcripts. Mean ± SEM from 5 to 6 independent experiments. *p < 0.05, **p < 0.01, ***p < 0.001 and ****p < 0.0001 with paired *t*‐test (M) and ANOVA plus Turkey's method (F–H, K).

To characterize the ubiquitination of Notch1, we predicted 17 potential deubiquitylase (DUBs) and 24 E3 ligase of Notch1 using the UbiBrowser database^23^ (Figure 4A), and identified UCHL1 as the most enriched DUBs in CSCs (Figure 4B,C). It was found that there was no significant difference in the mRNA expression levels of all E3 ligase and DUBs between CSCs and non‐CSCs including UCHL1. Western blot results also confirmed comparable UCHL1 protein levels in CSCs and non‐CSCs (Figure 4D). However, Immunoprecipitation results showed that compared to non‐CSCs, the binding between Notch1 and UCHL1 significantly increased in CSCs (Figure 4E). This indicated that the enhanced binding of Notch1 to DUBs UCHL1 resulted in the deubiquitination of Notch1 in CSCs, thus maintaining LCSC stemness. To confirm the crucial role of UCHL1 in regulating the ubiquitination of Notch1, we downregulated the expression of UCHL1 in CSCs (Figure 4F) and observed consistent downregulation of Notch1 protein using two independent shRNAs targeting UCHL1 (Figure 4G). The Notch1 protein level remained unaffected after knocking down the expression of another four enriched E3 ligases and deubiquitinases (DUBs): WWP1, MDM2, USP9X and PSMD7, which confirmed the specificity of UCHL1's effect (Figure 4H,I). Furthermore, UCHL1 knockdown efficiently increased the ubiquitination of Notch1 protein (Figure 4J).

Given that NO can increase Notch1 protein levels, we investigated the correlation between NO‐increased Notch1 protein and UCHL1. Notch1 protein expression was evaluated after UCHL1 knockdown in the presence of DETA NONOate. Results showed that DETA NONOate failed to reverse UCHL1 knockdown‐induced Notch1 degradation and increased ubiquitination (Figure 4K,L). Additionally, knockdown of UCHL1 also inhibited the expression level of stemness markers in LCSCs (Figure 4M). These findings indicated that NO‐instructed Notch1^high^ may be due to the UCHL1‐mediated protein deubiquitination, leading to maintenance of CSCs.

### 4.5 NO‐mediated S‐Nitrosylation of Notch1 facilitates its deubiquitination

**FIGURE 5:**
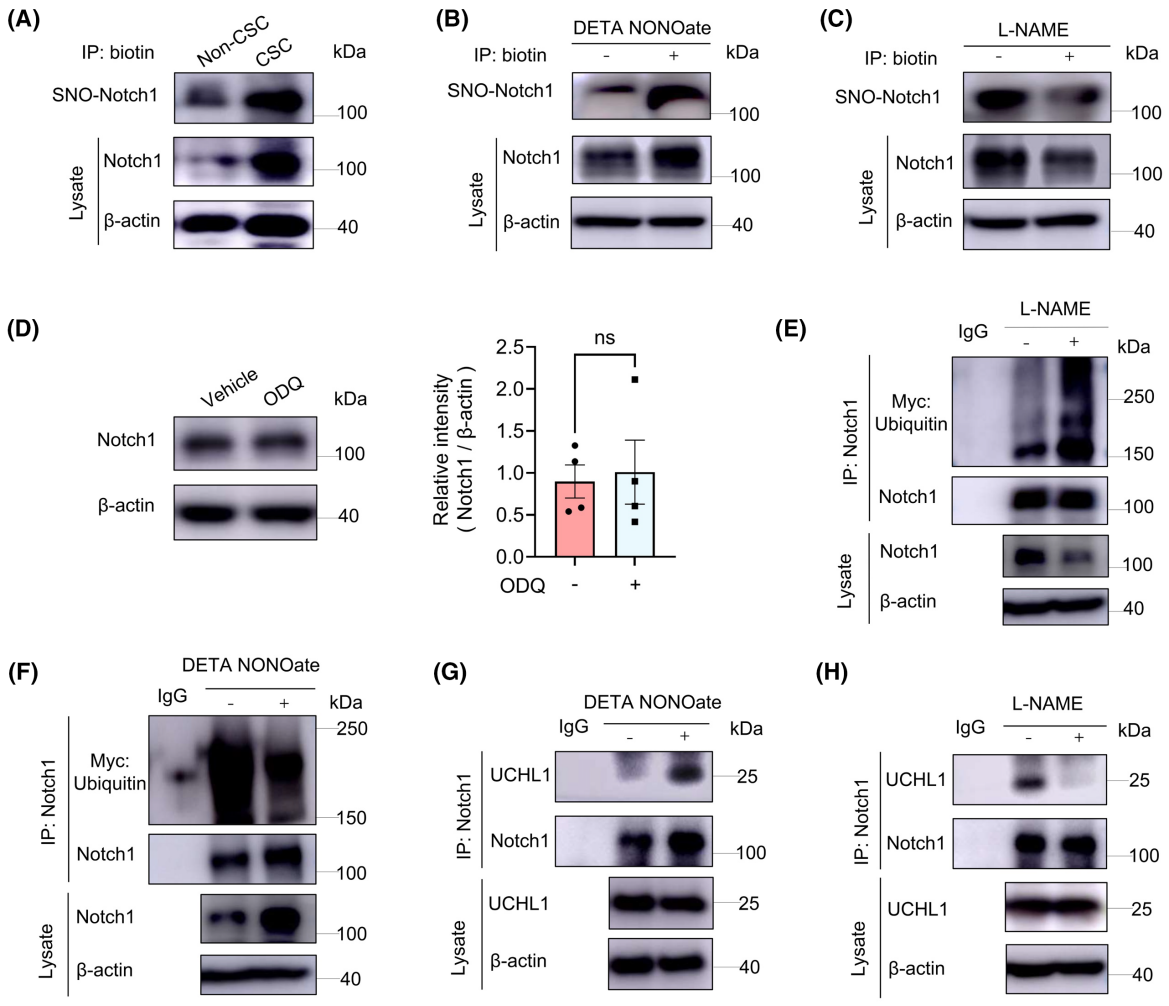
NO‐facilitated s‐nitrosylation of Notch1 promotes its binding to UCHL1. (A) Biotin switch assay was employed to detect the SNO‐Notch1 levels in both non‐CSCs and CSCs. (B) The SNO‐Notch1 level in DETA NONOate‐treated (20 μM, 24 h) CSCs were detected by biotin switch assay. (C) The SNO‐Notch1 level in L‐NAME‐treated (100 μM, 24 h) CSCs were detected by biotin switch assay. (D) Notch1 protein levels in CSCs with or without ODQ (10 μM, 24 h) were analysed by immunoblotting. Mean ± SEM from four independent experiments. (E) Immunoprecipitation analysis of Notch1 ubiquitination in CSCs in the presence or absence of L‐NAME (100 μM) for 24 h. (F) Immunoprecipitation analysis of Notch1 ubiquitination in CSCs treated with DETA NONOate (20 μM) for 24 h. (G) Co‐immunoprecipitation analysis of Notch1 and UCHL1 interaction in CSCs in the presence or absence of DETA NONOate (20 μM). (H) Co‐immunoprecipitation analysis of Notch1 and UCHL1 interaction in CSCs with or without L‐NAME (100 μM).

NO exerts its cellular effects through either activating the soluble guanylate cyclase (sGC)/cyclic guanosine 3′,5′‐monophosphate (cGMP) pathway or via S‐nitrosylation, a reversible posttranslational modification of Cys‐thiol groups.^18^, ^24^ Immunoprecipitation analysis revealed significantly enhanced S‐nitrosylation of Notch1 in CSCs compared to non‐CSCs (Figure 5A). Further results demonstrated that DETA NONOate increased the S‐nitrosylation of Notch1 in CSCs (Figure 5B), while L‐NAME decreased the S‐nitrosylation of Notch1 in CSCs (Figure 5C). Simultaneously, Notch1 protein level were upregulated in CSCs and DETA NONOate‐preincubated non‐CSCs but downregulated in L‐NAME‐treated CSCs. These results showed that NO‐mediated S‐nitrosylation of Notch1 inhibited its degradation in CSCs. In contrast, treatment with the sGC inhibitor ODQ did not alter Notch1 protein expression (Figure 5D), suggesting that NO‐induced enhancement of Notch1 protein is not mediated by the sGC‐cGMP signalling pathway but rather by Notch1 S‐nitrosylation.

To explore whether Notch1 S‐nitrosylation affects its ubiquitination, we examined the ubiquitination of Notch1 in response to DETA NONOate or L‐NAME. Immunoprecipitation results showed that L‐NAME promoted Notch1 ubiquitination, whereas DETA NONOate attenuated it (Figure 5E,F). This effect can be attributed to NO‐induced S‐nitrosylation of Notch1 and elevated binding of Notch1 to UCHL1, a process disrupted by L‐NAME (Figure 5G,H). In essence, excessive NO increases S‐nitrosylation of Notch1 protein in CSCs, facilitating its interaction with UCHL1. Reduced ubiquitination of Notch1 increased Notch1 protein expression levels, which ultimately promoting the stemness of LCSCs.

### 4.6 Targeting NO synthesis and UCHL1 restricts CSC pool in PDOs

**FIGURE 6:**
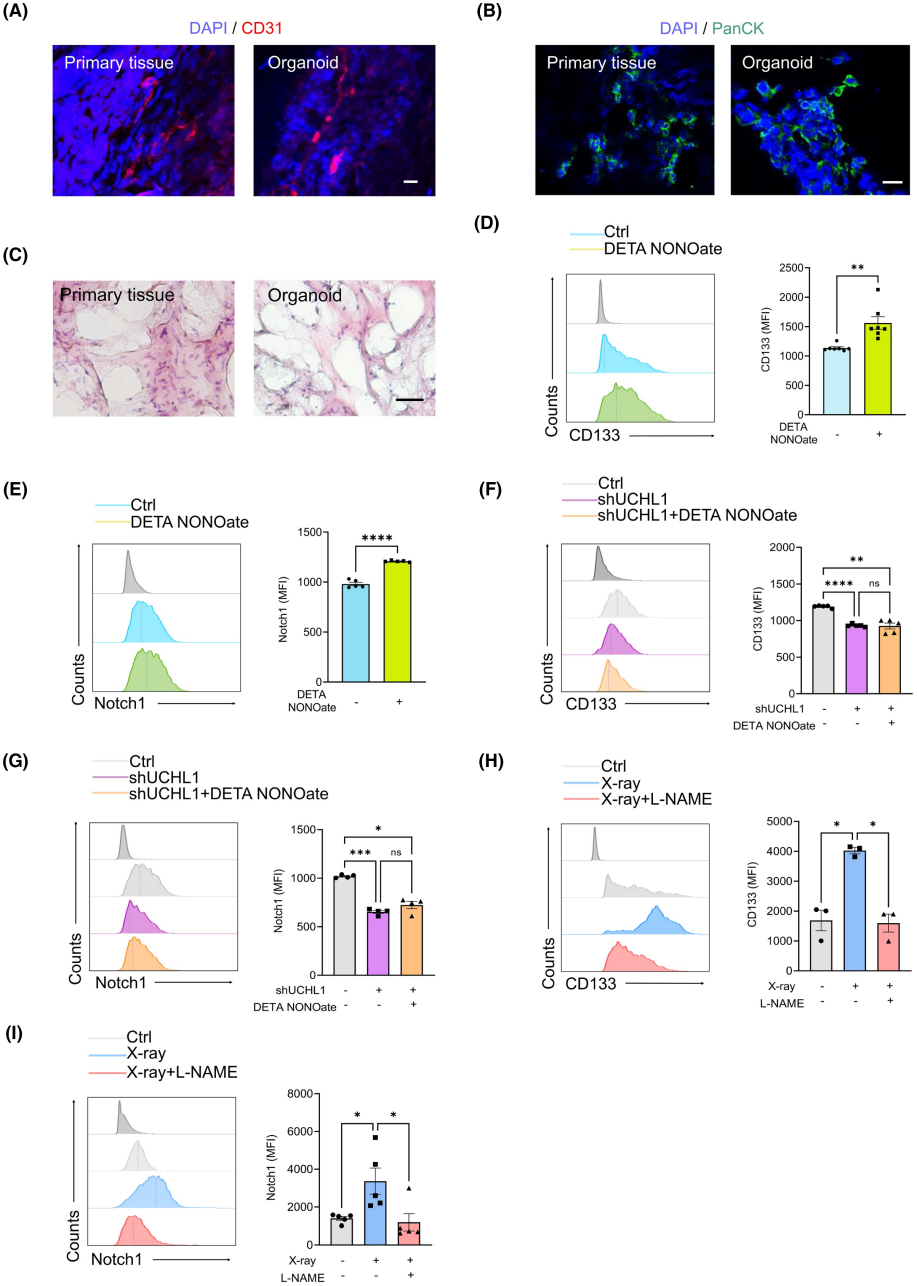
Targeting UCHL1 and NO downregulates the expression levels of Notch1 and CD133. (A) Representative of PDO‐primary tumour pair with immunostaining for CD31. Nuclei were stained with Hoechst. Scale bar, 20 μm. (B) Representative of PDO‐primary tumour pair with immunostaining for PanCK. Nuclei were stained with Hoechst. Scale bar, 20 μm. (C) Representative HE staining of PDO‐primary tumour pairs. Scale bars, 100 μm. (D, E) PDOs treated with or without DETA NONOate (20 μM) were analysed for CD133 and Notch1 protein. Representatives from six independent experiments. (F, G) PDOs were transfected with UCHL1 shRNAs in the presence or absence of the DETA NONOate (20 μM), followed by analysis of CD133 and Notch1 protein. Representatives from 4 to 5 independent experiments. (H, I) PDOs exposed to 10 Gy x‐rays were treated with L‐NAME (100 μM), followed by analysis of CD133 and Notch1 protein. Representatives from 3 to 5 independent experiments. *p < 0.05, **p < 0.01, ***p < 0.001, ****p < 0.0001 with paired *t*‐test (D, E) and ANOVA plus Turkey's method (F–I).

To evaluate the translational potential of targeting NO‐mediated Notch1 S‐nitrosylation and UCHL1‐instructed Notch1 deubiquitination, PDOs were established and evidenced by immunostainings of PanCK plus CD31 and H&E staining (Figure 6A–C). Immunofluorescence staining revealed PDOs with a vessel‐like structure and molecular marker expression similar to primary tumour tissues, confirming the successful organoid construction. In line with aforementioned results, treatment with DETA NONOate significantly increased the proportion of CD133‐positive tumour cells in NSCLC PDOs (Figure 6D), along with elevated expression of Notch1 protein (Figure 6E). Conversely, knockdown of UCHL1 led to a marked reduction in the proportion of CD133‐positive cells in PDOs (Figure 6F), accompanied by decreased Notch1 protein (Figure 6G). In addition, the reductions in CD133‐positive tumour cells and Notch1 protein in UCHL1‐knockdown PDOs could not be restored by DETA NONOate treatment (Figure 6F,G), confirming the Notch1 deubiquitylation and CSC maintenance as downstream consequences of NO metabolism.

Radiation therapy is a widely used treatment of human malignancies, but its efficacy is often compromised in patients with radiation‐insensitive tumours.^25^ The CSC theory provides a plausible explanation for radiation resistance.^26^ To assess the potential of our findings in addressing CSC‐mediated radiation resistance, we exposed PDOs to a single dose of 10 Gy radiation, resulting in a significant increase in the proportion of CD133‐positive tumour cells and Notch1 protein expression (Figure 6H,I). However, post‐radiotherapy treatment of the organoids with L‐NAME led to a decrease in both the proportion of CD133‐positive tumour cells (Figure 6H) and Notch1 protein levels (Figure 6I), indicating that NO regulation could control the CSC pool by targeting Notch1 protein in NSCLC PDOs.

## 5 DISCUSSION

Radiotherapy, utilizing high‐energy radiation, constitutes a pivotal treatment approach in the management of NSCLC, demonstrating substantial therapeutic efficacy.^26^, ^27^ The majority of patients diagnosed with NSCLC will undergo radiotherapy as part of their treatment regimen. However, resistance to radiotherapy in NSCLC presents a significant challenge, primarily attributed to a small subset of cells known as CSCs, awaken by radiation.^28^ These resilient CSCs exhibit augmented proliferative capabilities, ultimately driving cancer recurrence and metastasis.

CSCs exhibit resistance to radiotherapy due to multiple mechanisms. Notably, CSCs demonstrate heightened DNA repair capabilities compared to non‐CSCs, particularly through augmented homologous recombination and non‐homologous end joining pathways, which facilitate the repair of double‐strand breaks.^29^ Upon DNA damage induced by radiation, CSCs promptly engage DNA damage checkpoint response pathways, enabling swift repair of DNA lesions and mitigating cellular harm.^30^ Additionally, CSCs maintain reduced levels of reactive oxygen species (ROS), thereby sustaining an effective antioxidant defence system to counteract oxidative stress and minimize oxidative injury resulting from radiotherapy.^31^, ^32^ Furthermore, the dysregulated cell cycle dynamics of CSCs enable them to persist in the radioresistant G0/G1 phase, facilitating rapid post‐therapy proliferation.^33^ Moreover, interactions between CSCs and their microenvironment, involving endothelial and immune cells, also contribute to resistance, aiding CSCs in evading the impacts of radiotherapy.^34^, ^35^ Consequently, we propose an innovative strategy to surmount radiotherapy resistance in NSCLC by targeting CSC populations via modulation of NO metabolism.

NO is synthesized by nitric oxide synthase (NOS), using amino acid L‐arginine as the substrate. There are three isoforms: inducible NOS (iNOS), neuronal NOS (nNOS) and endothelial NOS (eNOS). Constitutive expression of nNOS and eNOS stably produces NO.^36^ In contrast, iNOS expression is induced by specific stimuli, leading to the generation of large amounts of NO. Of note, NO plays opposite roles depending on its concentration and context. On the one hand, local NO in tumour microenvironment (TME) is important for anti‐tumour immune response of CD8^+^ cytotoxic T cells; on the other hand, NO also activates pro‐tumour signals by maintaining CSC stemness.^37^ In this study, we employed RNA sequencing to demonstrate a significant enhancement of nitrogen metabolism in NSCLC CSCs. Importantly, we pinpointed a critical function of NO in maintain CSCs by regulating the Notch1 protein. However, precise mechanisms underpinning the elevated NO levels in CSCs remain unclear.

NO signalling is classified into the classical and non‐classical schemes. In the classical scheme, NO signalling is mediated through the activation of sGC, which convert GTP to cGMP. cGMP, in turn, activates cGMP‐dependent protein kinase (PKG) to lower the levels of potassium and calcium ions in the cytosol, leading to membrane hyperpolarization, neurotransmission, and vasodilation.^18^, ^38^ In contrast, the non‐classical scheme of NO signalling includes covalent post‐translational modification known as S‐nitrosylation. Cancer cells usually succumb to dysregulated levels of NO and S‐nitrosylation, contributing to malignant phenotypes, such as genomic instability, cell proliferation, anti‐apoptosis, angiogenesis and metabolic reprogramming.^24^ In this study, we uncovered that the S‐nitrosylation of Notch1 promoted the binding of Notch1 to UCHL1, thereby facilitating the deubiquitination of Notch1. In consistent, accumulating studies have assigned an essential role of Notch1 in maintaining CSCs. In our prior research, we have elucidated that enhanced mitophagy in CSCs resulted in mitochondrial DNA accumulation within lysosomes, triggering TLR9 activation and subsequent hyperactivation of Notch1. Notch1 interacted with AMPK to drive lysosomal AMPK activation, supporting CSC expansion.^8^ Similarly, CXCL2 activated the Notch1/Hey1 signalling pathway in breast cancer cells, leading to an increase in ALDH^+^ breast cancer stem cells.^39^ CircSLC4A7 interacted with HSP90 to regulate cellular stemness in gastric cancer by activating the Notch signalling pathway.^11^

Compared to patient‐derived cancer cell lines (PDCs) and patient‐derived tumour xenografts (PDXs), PDOs exhibit a high fidelity to the primary tumour and tremendous potentials for applications in personalized medicine.^40^, ^41^ In this study, we preformed translational research and confirmed that NO could regulate the stemness and the Notch1 protein in NSCLC PDOs. Of interest, L‐NAME reversed the enrichment of stemness in irradiated PDOs, supporting the idea that targeting NO may provide new strategies to address the radiotherapy resistance for NSCLC.

In summary, NO is enriched in CSCs of human NSCLC, driving the S‐nitrosylation of Notch1 protein and subsequently resulting in deubiquitylation of Notch1 protein. Accordingly, targeting NO synthesis and Notch1 deubiquitylation is efficient in overcoming the resistance of NSCLC to radiation therapy. These findings shed new light on the metabolic control of CSCs, providing novel clues for therapeutic explorations against human NSCLC.

## AUTHOR CONTRIBUTIONS

**Tenglong Zhang:** Data curation (equal); investigation (equal); methodology (equal); resources (equal); validation (equal); writing – original draft (equal). **Jiaxin Lei:** Conceptualization (equal); formal analysis (equal); investigation (equal); methodology (equal); validation (equal); writing – original draft (equal). **Ming Zheng:** Formal analysis (equal); investigation (equal); resources (equal). **Zhenke Wen:** Conceptualization (equal); funding acquisition (equal); project administration (equal); supervision (equal); writing – original draft (equal); writing – review and editing (equal). **Juying Zhou:** Conceptualization (equal); project administration (equal); supervision (equal); writing – review and editing (equal).

## CONFLICT OF INTEREST STATEMENT

The authors have no conflict of interest to declare.

## Supporting information


**Appendix S1:** Supporting Information.

## Data Availability

All data were included in the manuscript and could be obtained from the corresponding author upon reasonable request.
