# Supplementary material for: Nitric oxide facilitates the S‐nitrosylation and deubiquitination of Notch1 protein to maintain cancer stem cells in human NSCLC
Source: J Cell Mol Med. 2024 Nov 10;28(21):e70203. doi: 10.1111/jcmm.70203 (PMC11550923; doi:10.1111/jcmm.70203)
Supplement: Supplementary file 1 — Appendix S1: Supporting Information. [file JCMM-28-e70203-s001.pdf]

Supplementary Table 1 Clinical features of NSCLC patients

Demographic parameters	
No. of subjects	31
Sex (F/M)	13/18
Age (mean ± SEM [years])	59.7 ± 11.2
TNM staging (I/II/III)	15/16/0
Histological types	
Adenocarcinoma	27
Squamous carcinoma	4

Supplementary Table 2 Primers for qPCR

18srRNA	F	AGTCCCTGCCCTTTGTACACA
	R	GATCCGAGGGCCTCACTAAAC
CD133	F	GCACTCTATACCAAAGCGTCAA
	R	CTCCCATACTTCTTAGTTTCCTCA
SOX2	F	TACAGCATGTCCTACTCGCAG
	R	GAGGAAGAGGTAACCACAGGG
OCT4	F	ACATCAAAGCTCTGCAGAAAGAAC
	R	CTGAATACCTTCCCAATAGAACC
ALDH1A	F	CGCAAGACAGGCTTTTCAGAT
	R	CCCTCTCGGAAGCATCCA
NOS2	F	TTCAGTATCACAACCTCAGCAAG
	R	TGGACCTGCAAGTTAAAATCCC
PSEN1	F	GACGACCCCAGGGTAACTC
	R	ACTGACTTAATGGTAGCCACGA
PSEN2	F	AGTGTGTGATGAGCGGACG
	R	ACTGGGCAGTGTTCTCTCCAT
APH1A	F	TTTTTCGGCTGCACTTTCGTC
	R	TGCGACCAGGATGATAACGC
APH1B	F	CGAGCCGTTGCGTATCATCTT
	R	CCAAACAAGGGACGAAATCAGT
PSENEN	F	CTGGAGCGAGTGTCCAATGAG
	R	GCGCCAGACATAGCCTTTGAT
NCSTN	F	AATAAAACAGCTCCCTGTGTTCG
	R	ACTACGTGGATAACCCCTGTG
ADAM10	F	ATGGGAGGTCAGTATGGGAATC
	R	ACTGCTCTTTTGGCACGCT
ADAM17	F	GTGGATGGTAAAAACGAAAGCG
	R	GGCTAGAACCCTAGAGTCAGG
BAP1	F	GATACGTCCGTGATTGATGATGA
	R	TGAGTTGCACAAGAGTTGGGTA
USP18	F	AACGTGCCCTTGTTTGTCCAA
	R	GAGTCCTTCACCCGGATCGTA
USP25	F	GCACCAGCAGACGTTTTTGAA
	R	AGCATTCTTCGCAGTAAGGAAA
USP28	F	CACTGTTGCTACAGAACCATCT
	R	TGGGAGACTCCAGTAGACTCA
USP7	F	GGAAGCGGGAGATACAGATGA
	R	AAGGACCGACTCACTCAGTCT

Supplementary Table 2 Primers for qPCR

PSMD7	F	CCCAGCGGATCACAAACCA
	R	GCCTGTGGCGACTTTTTCC
USP8	F	GTCCAGGAGTCACTGCTAGTT
	R	AGGAGCCAGTTTTCATAGCCT
UCHL1	F	CCTGTGGCACAATCGGACTTA
	R	CATCTACCCGACATTGGCCTT
USP9X	F	AAGTGAAGCATGTCAGCGATT
	R	GCCACACATAGCTCCACCA
USP2	F	AGTTGAGAGATTACTGCCTCCA
	R	AAGAAAGCGAAGGAACTCCTG
CYLD	F	TCAGGCTTATGGAGCCAAGAA
	R	ACTTCCCTTCGGTACTTTAAGGA
BRCC3	F	GAGTCTGACGCTTTCCTCGTT
	R	TGTATCATCGTTCAACTCCCCT
USP1	F	ATGCCTGGTGTCATACCTAGT
	R	CAGTCCCACAAATGGTAACAAGT
USP4	F	GGACCCTTAATGAGGACCACA
	R	TTCCACTGCTTGAACCACCG
USP15	F	CGACGCTGCTCAAAACCTC
	R	TCCCATCTGGTATTTGTCCCAA
USP11	F	CGTTTCCGGGACCAGAATCC
	R	CATCGCCGTCCGTTCTCTTC
USP5	F	GCTGCTGTCAGTATTACCGAC
	R	AAAGCCCAGAAACGTGTTCATA
FBXW7	F	GTGAAGCTGGTGGAAGAGAAG
	R	TGCTCAGGCACGTCAGAAAAG
ITCH	F	AGCGTAGTCAGCTTCAAGGAG
	R	AGGTGGCAATGGACCAAGAG
MDM2	F	GAATCATCGGACTCAGGTACATC
	R	TCTGTCTCACTAATTGCTCTCCT
NEDD4	F	CTTTATCCATTACCGACAG
	R	GGTGGCTTCATCTTCTC
FBXW11	F	CCGACTCGGTGATTGAGGAC
	R	CCGATGTTCCCCACATCCAA
MIB1	F	CAGCCAGAGGAATCTTTGCAG
	R	CCTTTCCCCTACGTCCATTTC

Supplementary Table 2 Primers for qPCR

NEDD4L	F	GACATGGAGCATGGATGGGAA
	R	GTTCGGCCTAAATTGTCCACT
SMURF1	F	GCTTCAAGGCTTTGCAAGGTT
	R	TGGGAGCCACCAACAAAAGT
SMURF2	F	GTGCTGGATTTCTCGGTTGT
	R	CCTCCTGTGCCTATTCGGT
HECW2	F	AAATCCCCAGATGCGGTACAC
	R	CGGCTCTCAGAAGTCACCA
BTRC	F	TGGCTCATCTGACAACACTATC
	R	CGAATACAACGCACCAATTCC
MIB2	F	ACCTGCTGCTGTACGACAAC
	R	GTGCATGTAGCACTGCGTG
MYLIP	F	GCAGGCGACTGGGAATCATAG
	R	CGGTTTCTCAGGTTTAGCCAT
ARIH1	F	GCCGGACGATGATACCCTG
	R	TCGTAGCGGTAATCCTCCTCC
WWP1	F	TTGCTGAGCTCATGGGAAGT
	R	TGGTGGTAGATCCAAGCGAT
WWP2	F	TGGAAGGCGGAAGTAGGA
	R	GTGAAGCTGGTGGAAGAGAAG
RBCK1	F	TTGACAACACCTACTCGTGC
	R	TTGACGTGGAAACACACAGGG
UBOX5	F	GCCTCATCTAGCAGAGTGTCT
	R	GTGGCTAAACACCACTTGGCT
BARD1	F	GGTATCCTTCTGTAGCCAACCA
	R	GGAGCCACTTGCTAGTAAGTCT
PRKN	F	AAATGCCCAGACAAGATGCC
	R	GGCCTCTCACGACTGAGTT
FBXL2	F	TGGAACATCTTAGCCCTGGAT
	R	CCACCACTCGACCCTCTACAT
RNF146	F	AAACAGGAAAGCGAACGAGTC
	R	GTTTGCAGACAAATGGCACAT
FBXW8	F	TGCCTTACGAATTGGCAATCA
	R	TGCGATTCTTCCAGTTGGTT
FBH1	F	ATGAGACGGTTTAAGCGGAAG
	R	ATGGTTCGGATCTCTGTTTGTC
NOTCH1	F	GAGGCGTGGCAGACTATGC
	R	CTTGTACTCCGTCAGCGTGA
